# Shared resistome and mobilome signatures across One Health *Escherichia coli* sources

**DOI:** 10.3389/fbinf.2026.1930054

**Published:** 2026-10-21

**Authors:** Eberechi Phoebe Nnah, Arshad Ismail, Akebe Luther King Abia, Sabiha Essack, Daniel Gyamfi Amoako

**Affiliations:** 1 Antimicrobial Research Unit, School of Health Sciences, University of KwaZulu-Natal, Durban, South Africa; 2 Sequencing Core Facility, Division of the National Health Laboratory Service, National Institute for Communicable Diseases, Johannesburg, South Africa; 3 Environmental Research Foundation, Durban, South Africa; 4 School of Pharmacy, University of Jordan, Amman, Jordan; 5 Department of Pathobiology, University of Guelph, Guelph, ON, Canada

**Keywords:** abundance, antibiotic resistance genes, diversity, *Escherichia coli*, metagenomics, mobile genetic elements, One Health, resistome

## Abstract

**Background:**

Antimicrobial resistance (AMR) is a major One Health challenge facilitated by the persistence and dissemination of antibiotic resistance genes (ARGs) and mobile genetic elements (MGEs) across interconnected human, animal, and environmental compartments. Despite growing recognition of environmental reservoirs in AMR ecology, integrated resistome and mobilome studies across One Health interfaces remain limited in Africa. This study applied shotgun metagenomic sequencing to culture-derived pools of multidrug-resistant (MDR) *E. coli* to characterize resistome composition and mobilome signatures across human, animal, and environmental sources in uMgungundlovu District, South Africa

**Methods:**

A total of 539 MDR *E. coli* isolates recovered from human, food-animal, wastewater, and other environmental sources were grouped into 20 source-specific pools. ARGs, plasmid-associated sequences, and MGE-associated sequences were annotated against the Comprehensive Antibiotic Resistance Database, ACLAME, and MetaStorm MGE database, respectively. Analyses included alpha diversity, Bray–Curtis dissimilarity, clustering, ordination, and permutational multivariate analysis of variance (PERMANOVA); MetaCompare prioritized pools using ARG–MGE–pathogen co-occurrence patterns.

**Results:**

Across the 20 pools, 247 distinct ARGs were detected, of which 86 (35%) occurred in at least one pool from each of the three sectors. Antibiotic inactivation and target protection were the most highly represented resistance mechanisms. ARG richness, Shannon diversity, and Simpson diversity did not differ significantly among sectors. Hierarchical clustering and ordination demonstrated substantial cross-sector overlap and within-sector heterogeneity. Nevertheless, PERMANOVA identified a significant but modest association between source sector and resistome composition (pseudo-F = 1.585, *R* ^2^ = 0.157, *p* = 0.022), with no significant evidence of unequal multivariate dispersion (*F*_2_,_17_ = 2.498, *p* = 0.086). Of 1,012 plasmid-associated features, 713 (70%) occurred in at least one pool from each sector, while 232 of 975 MG E-associated features (24%) were shared. Exploratory random forest classification did not significantly outperform classification under permuted source labels (*p* = 0.053).

**Conclusion:**

These findings reveal modest sector-associated resistome structuring alongside substantial cross-sector overlap in ARG, plasmid-associated, and MGE-associated signatures. Although the pooled data do not establish direct transmission, active mobility, or physical ARG–MGE linkage, they identify shared resistome patterns and source groups that warrant targeted, isolate-resolved and linkage-aware investigation within integrated One Health surveillance.

## 1 Introduction

Antimicrobial resistance (AMR) is a major threat to human, animal, and environmental health because it reduces the effectiveness of antimicrobial therapy and increases the risk of persistent, difficult-to-treat infections. Recent global estimates indicate that bacterial AMR was directly responsible for 1.14 million deaths and associated with approximately 4.71 million deaths globally in 2021, highlighting its substantial public health burden (Naghavi et al., 2024). The continued emergence of multidrug-resistant bacteria further limits therapeutic options in both clinical and veterinary settings (Aslam et al., 2018). Resistant bacteria and antibiotic resistance genes (ARGs) can persist across interconnected ecological compartments, including wastewater (Hendriksen et al., 2019), clinical settings, livestock production systems, surface water, agricultural soils, and food-processing environments (Pal et al., 2016; Rousham et al., 2018). Within this continuum, *E. coli* is an important One Health sentinel organism because it is found in both human and animal intestinal microbiota and is frequently recovered from food-production environments, wastewater, agricultural soil, and surface water. Its occurrence in these environments, combined with well-characterized intrinsic and acquired resistance mechanisms, enables the comparison of antimicrobial resistance patterns from human, animal, and environmental sources. However, *E. coli* should be regarded as an indicator organism rather than a complete representation of the bacterial communities or resistomes present in each environment (Bondo et al., 2016; García-Béjar et al., 2021; Gaşpar et al., 2021; Jain et al., 2021; Montealegre et al., 2018; Mukherjee et al., 2021; Nji et al., 2021; VinodhKumar et al., 2019).

The spread of antimicrobial resistance is strongly influenced by mobile genetic elements (MGEs), including plasmids, transposons, integrons, recombinases, insertion sequences, and other mobilizable elements. These elements facilitate the acquisition, maintenance, and redistribution of resistance determinants across bacterial populations and ecological interfaces (Jian et al., 2021; Zhang et al., 2021). Consequently, resistome analysis is increasingly strengthened by parallel assessment of mobile genetic element and plasmid-associated signatures, particularly in One Health studies where human, animal, and environmental reservoirs are sampled within the same geographic region (Collignon and McEwen, 2019; Oh et al., 2018).

A One Health framework is especially relevant in settings where human settlements, intensive animal production, agriculture, wastewater systems, and surface-water networks are spatially connected. In such systems, wastewater discharge (Hendriksen et al., 2019), antimicrobial use, manure application, slaughter activities, and environmental runoff may create overlapping selective pressures and opportunities for resistant bacteria or resistance determinants to persist across compartments (Collignon and McEwen, 2019; Rousham et al., 2018). However, in many low- and middle-income settings, integrated genomic and bioinformatic surveillance of antimicrobial resistance across human, animal, and environmental sources remains limited (Rousham et al., 2018).

Bioinformatics workflows that combine functional annotation, diversity analysis, ordination, feature ranking, and resistome-risk prioritization provide a useful framework for comparing resistance profiles across complex sample sets (Arango-Argoty et al., 2016; Ekwanzala et al., 2020; Oh et al., 2018; Sudarikov et al., 2017). In particular, ARG-targeted sequencing and pooled-source analysis can provide a broad overview of resistance-gene diversity across multiple source categories when isolate-resolved whole-genome sequencing of all available isolates is not feasible (Leonard et al., 2018; Noyes et al., 2017). However, such approaches require careful interpretation because pooled datasets do not provide isolate-level prevalence estimates, strain-resolved genotypes, or direct evidence of gene co-carriage within individual genomes.

This study addresses the limited number of integrated, district-level AMR surveillance data from human, animal, and environmental sources in South Africa. Its innovative contribution is the combined evaluation of the total resistome, the acquired ARG fraction, intrinsic and chromosomal resistance determinants, plasmid-associated features, MGE-associated features, resistome diversity, model-based ARG discrimination, and MetaCompare risk prioritization within a geographically linked dataset of pooled MDR *E. coli* source groups. By distinguishing acquired ARGs from chromosomal and intrinsic determinants and interpreting plasmid- and MGE-associated matches separately from confirmed mobile elements, the study provides a cautious framework for extracting surveillance information from archived isolates when isolate-resolved sequencing is not feasible.

## 2 Materials and methods

### 2.1 Ethical approval

This study is part of a larger research program on Antibiotic Resistance and One Health. The Biomedical Research Ethics Committee (reference: BCA444/16) and the Animal Research Ethics Committee (reference: AREC/007/018) at the University of KwaZulu-Natal and the KwaZulu-Natal Provincial Health Ethics Committee (NHRD Reference: HRKM108/17KZ_2017RP3_437) provided ethical approval. The Department of Agriculture, Forestry, and Fisheries (DAFF) granted a Section 20A permit to conduct research on animals (Ref. 12/11/15). Gatekeeper’s permission was obtained from Umgeni Water to sample the water. The KwaZulu-Natal Department of Health, the uMgungundlovu District Manager, and health facility managers granted access to the healthcare facilities and data, and the National Health Laboratory Service (NHLS) provided access to microbiology data.

### 2.2 Study area and sample sources

This study was carried out in the uMgungundlovu District of KwaZulu-Natal Province, South Africa. The district has an estimated population of 1.10 million (1,095,865), accounting for about 10% of the provincial population. The district has a mix of urban and rural areas, with three major habitation typologies (rural farming, traditional, and urban), as well as intensive food-animal production and crop cultivation near water bodies (Makhaye et al., 2021).

We examined MDR *E. coli* isolates from humans, animals, and the environment, which represented three One Health sectors. The human-source collection consisted of 12 isolates obtained from clinical specimens associated with bloodstream infections (BSIs; *n* = 7) and urinary tract infections (UTIs; *n* = 5) processed by the National Health Laboratory Service. The animal-source collection included 16 poultry-associated isolates and 328 pig-associated isolates recovered from samples taken on farms, at abattoirs, and at food-processing or retail facilities. These included fecal and caecal samples, carcass swabs or rinsates, and retail meat samples. The environmental-source collection included isolates from 37 soil and litter fertilizer samples, as well as 146 wastewater and surface-water samples, including wastewater influent and effluent, and river samples collected upstream and downstream of discharge. In total, 539 MDR *E. coli* isolates were included (Supplementary Table S1).

The isolates were obtained from previously conducted source-specific studies. Pig-associated isolates were derived from the longitudinal farm-to-fork investigation reported by Abdalla et al. (2021). Poultry-associated isolates were obtained from the poultry-production study conducted by McIver et al. (2020). UTI-associated isolates were obtained from the study reported by Kubone et al. (2020), while BSI-associated isolates were obtained from the clinical investigation reported by Hetsa et al. (2025). Soil and litter-associated isolates were obtained from Fatoba et al. (2022) agricultural investigation, while wastewater and associated surface-water isolates were obtained from Mbanga et al. (2021). The isolates were collected between August 2017 and February 2019. They had previously been identified and tested for antimicrobial susceptibility in their respective source studies.

The two human pooled source groups (pools) were labeled UTI and BSI. The animal pooled source groups included chicken retail meat (CRM), chicken litter (CL), pig retail meat (PRM), and longitudinal pig-fecal pools collected in October (PFO1-PFO3), November (PFN), December (PFD1-PFD2), and January (PFJ). The environment-related pools included wastewater influent (Inf), wastewater effluent (Eff), upstream river water (US), downstream river water (DS), soil after manure application (SaM), litter fertilizer (LF), pig wastewater (PWW), and chicken wastewater (CWW).

### 2.3 Pooling strategy, DNA extraction, library preparation, and sequencing

Sample preparation was similar to Leonard et al. (2018), with slight adjustments. In brief, stored *E. coli* isolates were resuscitated in Tryptic Soy Broth and incubated at 37 °C for 24 h. Afterward, each culture was streaked onto selective media, Eosin Methylene Blue agar, to confirm presumed *E. coli* morphology. A well-isolated individual colony was then subcultured on nutrient agar to yield pure bacterial colonies. One pure, distinct colony from each isolate in the same predefined source category, selected with sterile tips, was then combined into microcentrifuge tubes to create a pooled source-group preparation. See Supplementary Table S1 for more information on the pooling strategy. DNA was extracted from each pooled preparation using the GenElute Bacterial Genomic DNA Kit (Sigma-Aldrich, St. Louis, MO, United States of America) according to the manufacturer’s directions. DNA concentration and purity were determined using a NanoDrop 1,000 spectrophotometer (Thermo Scientific, Wilmington, DE, United States of America). DNA concentrations ranged between 30.60 and 94.60 ng/μL, with a mean of 56.68 ng/μL. A260/A280 ratios ranged between 1.68 and 1.88. Supplementary Table S2 provides pool-level DNA concentration and purity values. Sequencing libraries were prepared using the Nextera XT DNA Library Preparation Kit (Illumina, San Diego, CA, United States of America) and sequenced as paired-end reads on an Illumina NextSeq 500 platform at the Sequencing Core Facility of the National Institute for Communicable Diseases (NICD) in Johannesburg, South Africa. Thus, the sequencing input was a pooled isolate preparation from an *E. coli* culture, which reduced background DNA from non-target taxa and increased the sensitivity of ARG detection (Noyes et al., 2017). Therefore, rather than representing unbiased whole-metagenomic abundance, normalized abundance patterns should be interpreted as *E. coli* culture genomic profiles.

### 2.4 Bioinformatics processing and functional annotation

MetaStorm was used to process the sequenced data (Arango-Argoty et al., 2016; Ekwanzala et al., 2020; accessed 18 September 2023). Adapter sequences and low-quality bases were removed using Trimmomatic v0.36 with the default parameters implemented in MetaStorm. Quality-filtered reads were assembled using Meta-IDBA-UD v1.1.3, and genes were predicted from the assembled scaffolds using Prodigal v2.6.3 in metagenomic mode. ARG abundance was scaled using 16 S rRNA marker-gene detection via BLASTN, with an E-value threshold below 1 × 10^−10^ and an identity threshold above 90%. This marker-based scaling was used to account partly for variations in sequencing depth and recoverable bacterial DNA across pooled source-group libraries. It should not be interpreted as a community-wide estimate of ARG abundance, as the libraries were derived from cultured *E. coli* pools rather than from complex microbial communities. Differences in 16 S recovery, assembly efficiency, sequencing depth, marker-gene copy number, and unequal contributions of isolates to the pools may all affect the resulting values. The normalized values were thus used for relative comparisons within this dataset rather than absolute ARG copy-number estimates. Some resistance annotations were named after determinants found in non-*E. coli* taxa, such as the *ACT, OXY, Mex, and Vga* families. However, because the annotation was based on sequence similarity, these labels could represent homologous *E. coli* sequences, distant database matches, or contamination-introduced sequences. The taxonomic assignment of ARG-containing scaffolds, complete gene coverage, genomic context, and independent confirmation were not examined. Predicted genes were queried against Comprehensive Antibiotic Resistance Database v3.1.0 (BLASTX; E-value < 1e-10; identity >90%; minimum alignment length >25 aa) to identify ARGs. Plasmid-associated features were detected using ACLAME plasmid annotations v0.4 (BLASTN; E-value < 1e-10; identity >90%), and mobile genetic element features were profiled using the MetaStorm MGE database v1.0. ARG presence was defined as a normalized value greater than zero in a pooled source group.

### 2.5 Resistome composition, clustering, diversity, and ordination analysis

ARG profiles were summarized by gene, drug class, and resistance mechanism. A heatmap (Sudarikov et al., 2017) was generated to display the most abundant ARGs. Each ARG in the heatmap has a normalized *Z*-score across pools (mean = 0, SD = 1) to indicate relative enrichment or depletion in each pooled source group. Distance-based clustering was used to hierarchically cluster pools and ARGs, which were then displayed as a clustered heatmap with annotations for the source sector. Ward linkage was used to perform hierarchical clustering based on Euclidean distances among *Z*-scored ARG relative abundances. ARGs were grouped by antibiotic class and resistance mechanism. Where an ARG was associated with more than one class or mechanism, it was assigned only once based on the primary CARD annotation returned by the pipeline. This prevented multiple counts of the same ARG in proportional summaries. The distributions of total ARG relative abundance were displayed as boxplots per sector, with sample IDs superimposed. The union and intersections of ARGs, plasmids, and MGEs across sectors were determined using a binary presence/absence matrix, and the results were displayed in a Venn diagram. To compare resistome composition across pools, a distance matrix was generated using normalized ARG profiles and used to perform hierarchical clustering. Ward’s minimum-variance approach was used to construct the dendrogram. Ward’s approach is conventionally specified for (squared) Euclidean distances, and it reduces within-cluster variation at each agglomeration stage.

Alpha-diversity (Sudarikov et al., 2017) of ARG profiles was calculated for each pooled source group from normalized ARG abundances using the following criteria: (i) richness (number of ARGs detected in a pooled source group), (ii) Shannon diversity (accounts for richness and abundance distribution), and (iii) Simpson diversity. The pools were divided into three sectors (human, animal, and environment), and boxplots were used to depict sector-level distributions with points labeled by pool source. For beta-diversity (Sudarikov et al., 2017) and ordination, Bray-Curtis distances were calculated using the relative-abundance ARG matrix to quantify compositional dissimilarity between pools. The Bray-Curtis distance matrix was displayed using the following methods: principal coordinate analysis (PCoA) of Bray-Curtis, NMDS of Bray-Curtis. Pools were color-coded by sector (human/animal/environment) and overlaid with sector ellipses to show within-group dispersion.

### 2.6 Feature importance analysis

Random Forest was used as an exploratory supervised feature-ranking approach to identify ARGs contributing to discrimination among pooled *E. coli* resistome profiles from human, animal, and environmental sectors. The analysis used the 20 pooled source-group profiles as independent observations. The normalized ARG relative-abundance matrix was used as the predictor dataset, with One Health sector (human, animal, or environment) specified as the classification outcome. ARG features with no variation across the analyzed pools were excluded before model fitting, leaving 102 informative ARG predictors for the final Random Forest analysis. No additional rare- or low-abundance filtering was applied. The primary models were fitted using 1,000 trees, with prespecified random seeds used to ensure reproducibility.

Model performance was evaluated using repeated five-fold cross-validation with 10 repeats. Stratification was applied where feasible; however, because only two human pools were available, representation of the human group in every validation fold was not possible. The number of candidate variables considered at each split (*mtry*) was tuned across candidate values, with the best-performing setting selected according to cross-validated classification accuracy. To determine whether the observed classification performance exceeded that expected by chance, a label-permutation test was performed by randomly shuffling the human, animal, and environmental source labels 999 times while retaining the ARG predictor matrix unchanged. To reduce computational demand while maintaining a like-for-like comparison, the permutation analysis used 100-tree Random Forest models for both the observed-label model and each permuted-label model, with *mtry* fixed at the value selected during model tuning. The separate 1,000-tree cross-validation estimate was not used to calculate the permutation *p*-value. The corrected Monte Carlo permutation *p*-value was calculated as (*b*+1)/(B+1), where *b* was the number of permuted accuracies equal to or greater than the tree-matched observed accuracy and B = 999 was the total number of permutations.

Feature importance was quantified using Mean Decrease Gini, which measures the contribution of each predictor to improvements in node purity across the fitted decision trees. The 35 ARGs with the highest Mean Decrease Gini values were retained for visualization. Feature-ranking stability was additionally evaluated across 100 repeated Random Forest analyses. In each run, a stratified 75% training subset comprising 15 pools was generated, with the remaining five pools retained for testing. A 1,000-tree Random Forest was fitted to each training subset, and the 35 highest-ranked ARGs were recorded. Stability was expressed as the percentage of repeated runs in which each ARG occurred among the top 35 predictors; no arbitrary binary stability threshold was imposed.

### 2.7 Resistome-risk analysis

Resistome risk was estimated using MetaCompare. MetaCompare is a computational pipeline that ranks resistome risk based on sequence-level co-occurrence patterns involving ARGs, MGEs, and human-pathogen-associated sequences. Resistome-risk classes categorize ARG sequences of interest based on their likelihood of mobilizing ARGs to bacterial infections and causing harm to human health, enabling the prioritization of mitigation activities (Oh et al., 2018). The original MetaCompare pipeline was used to compare the assembled scaffolds for each pooled source group separately. The MetaCompare analysis employed ResFinder for acquired ARG annotation, ACLAME for MGE-associated annotation, and BV-BRC for human-pathogen-associated matches. The ResFinder analysis was carried out separately from the CARD-based resistome analysis described above; CARD annotations were not used to replace the ResFinder component of MetaCompare. The pool-level resistome-risk values were obtained directly from the final MetaCompare risk-score output, without manual calculation, using an *e*-value below 1 × 10^−10^, sequence identity above 60%, and minimum alignment length above 150 bp. Sector-level estimates were computed using unweighted arithmetic means of pool-level scores within each sector. Because MetaCompare was designed for assembled metagenomic datasets, its use with culture-derived *E. coli* pools was considered exploratory. Sequencing depth, assembly completeness, the number and composition of isolates contributing to each pool, and partial sequence recovery can all influence the score. The 60% identity threshold may include distant homologs that are not functionally equivalent to the reference determinant. Complete gene coverage, reference sequence coverage, sequence completeness or structural integrity, and confirmation with an independent database were not evaluated. As a result, the scores were interpreted as comparative within-dataset prioritization measures rather than direct probabilities of clinical risk, functional mobility, or pathogen transmission.

### 2.8 Statistical analysis

Bray–Curtis dissimilarities were calculated from the ARG relative-abundance matrix. Differences in resistome composition among the human, animal, and environmental sectors were assessed using permutational multivariate analysis of variance (PERMANOVA) implemented with the adonis2 function in the vegan package in R. The model included sector as the explanatory variable and was evaluated using 999 unrestricted permutations. Kruskal–Wallis tests were used to compare alpha-diversity indices among sectors. Where appropriate, Dunn’s pairwise tests were performed, and p-values were adjusted using the Benjamini–Hochberg false-discovery-rate procedure. Statistical significance was determined at *α* = 0.05. Homogeneity of multivariate dispersion among sectors was evaluated using PERMDISP based on distances to group centroids, with significance assessed using 999 permutations.

## 3 Results

### 3.1 Sequencing output and pooled source-group structure

The sequencing of the 20 pooled *E. coli* source groups yielded more than 23 GB of uncompressed data. The number of raw reads per pool varied between 2,724,000 and 6,816,807, with a mean of 5,766,677 and a standard deviation of 840,627. After quality control, 2,668,207 to 6,684,895 reads per pool were retained, representing an average of 97.93% of the raw reads. Assemblies contained 13,702 to 154,416 scaffolds per pool. Pool-level sequencing, quality control, scaffold count metrics, and MetaCompare risk values are presented in Supplementary Table S3.

### 3.2 ARG composition and relative abundance profiles

Across the 20 pooled source groups, 247 distinct ARGs were detected. Efflux- and inactivation-associated determinants were highly represented. Hierarchical clustering did not produce clear separation by sector, with pooled source groups from different sectors occurring within the same dendrogram branches (Figure 1). This pattern indicates limited sector-level separation of ARG relative-abundance profiles under the clustering approach used. PFO1 showed visually elevated signals across several ARG clusters; however, it was not identified as a statistical outlier and should not be interpreted as evidence of a targeted surveillance priority based on the heatmap alone. Origin-class analysis showed that chromosomal determinants accounted for the largest proportion of the resistome signal across all three sectors, representing 79.67% in animal, 81.07% in environment, and 85.82% in human pools (Figure 2a). Intrinsic determinants accounted for a further 3.52%, 3.89%, and 3.15%, respectively. The acquired fraction was comparatively smaller but was higher in animal (16.82%) and environmental (15.04%) pools than in human pools (11.03%). Chromosomal and intrinsic determinants, including efflux systems, porins, and regulatory loci, were considered separately from acquired ARGs to distinguish their contribution from determinants more commonly associated with horizontal transfer.

**FIGURE 1:**
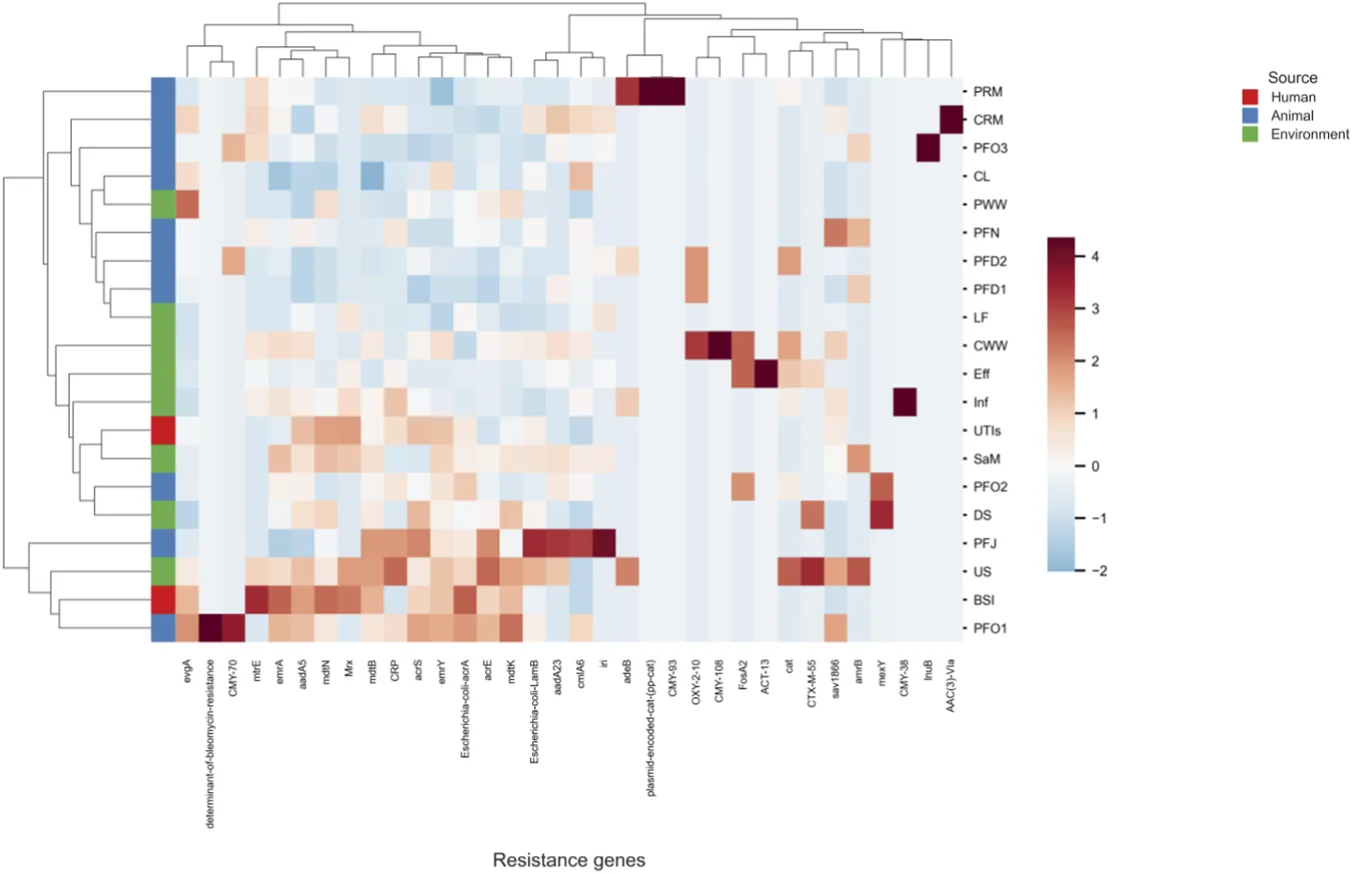
Clustered heatmap of the most abundant ARGs across sources. ARG relative abundances were Z-score standardized for visualization. Hierarchical clustering of samples and ARGs reveals shared resistomes across human, animal, and environmental sectors, with source sectors indicated by the top annotation bar.

**FIGURE 2:**
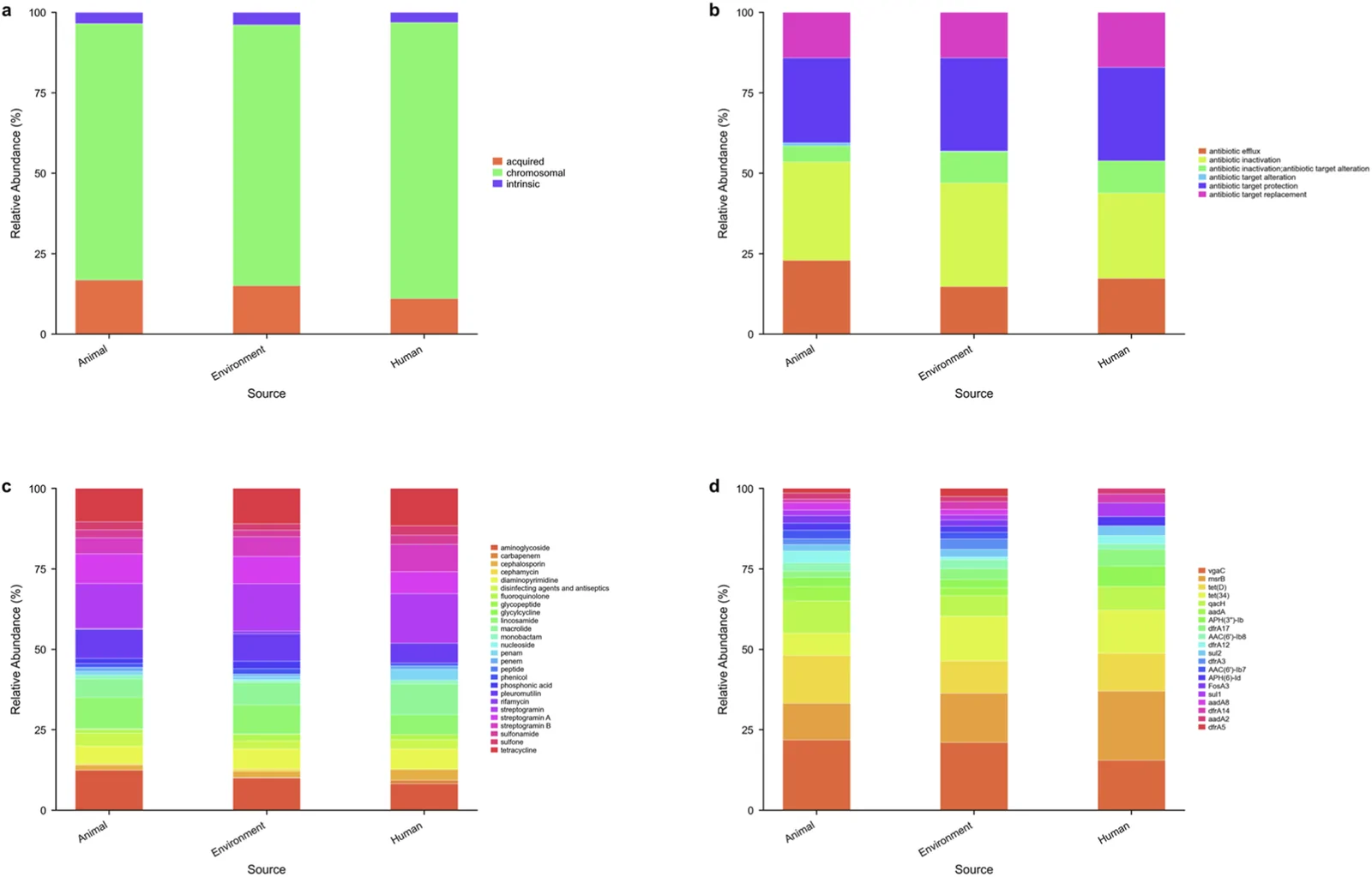
Sector-level composition of the *Escherichia coli* resistome: **(a)** ARG origin; **(b)** acquired ARG resistance mechanisms; **(c)** acquired ARG drug classes; and **(d)** the 20 most abundant acquired ARGs. Bars show the relative contribution (%) of the categories displayed within each sector and sum to 100%.

All three sectors contained multiple resistance mechanisms, including antibiotic efflux, antibiotic inactivation, target alteration, target protection, and target replacement (Figure 2b). Antibiotic inactivation and target protection were the largest contributors across sectors, accounting for 30.58% and 26.38% in animal, 32.13% and 28.89% in environment, and 26.55% and 29.05% in human pools, respectively. Target replacement accounted for 14.15%, 14.18%, and 17.13% in animal, environment, and human pools, respectively. Acquired ARGs collectively represented resistance to a broad range of antimicrobial classes, with substantial contributions from streptogramins, tetracyclines, aminoglycosides, macrolides, lincosamides, diaminopyrimidines, sulfonamides, phenicols, and disinfecting agents and antiseptics (Figure 2c). Among the most abundant acquired ARGs, *vgaC*, *msrB*, *tet(D)*, *tet(34)*, and *qacH* were consistently prominent, although their relative contributions varied among sectors (Figure 2d). *vgaC* accounted for 21.77%, 21.08%, and 15.52% of the displayed acquired ARG signal in animal, environmental, and human pools, respectively. Other highly represented determinants included *aadA, APH(3″)-Ib, dfrA17, AAC(6′)-Ib* variants, *dfrA12, sul2, dfrA3, APH(6)-Id, FosA3, sul1, aadA8, dfrA14, aadA2,* and *dfrA5*. These determinants encompass antibiotic inactivation, target protection, target replacement, and acquired efflux mechanisms, demonstrating a diverse acquired resistome across the sampled sectors.

Total ARG relative-abundance distributions overlapped substantially across sectors (Supplementary Figure S1). Human pools were positioned on either side of the sector median, with BSI above and UTI below the median. Animal pools showed the greatest upper-range variation, with PFJ and PFO1 displaying the highest relative-abundance values, while most remaining animal pools occurred near or below the sector median. Environmental pools also spanned both sides of the median, with US showing the highest relative abundance, whereas several environmental pools occurred below the median.

### 3.3 Shared and unique ARGs

Of the 247 distinct ARGs, 86 (35%) were detected in at least one analyzed pool from each of the human, animal, and environmental sectors (Supplementary Figure 3a). This was an intentionally broad, pool-level definition of shared detection and does not imply that an ARG was prevalent across a sector or present in every pool within that sector. The largest pairwise-exclusive overlap was between the animal and environmental sectors, comprising 59 ARGs (24%). One ARG (<1%) was exclusive to the human-animal pair, while 8 ARGs (3%) were exclusive to the human-environment pair. ARGs uniquely detected within the analyzed pools comprised five human-only (2%), 47 animal-only (19%), and 41 environment-only (17%) ARGs (Supplementary Table S5). However, absence from the other analyzed pools should not be interpreted as evidence of biological sector specificity.

### 3.4 ARG clustering, alpha diversity, and beta diversity

Hierarchical clustering based on Bray–Curtis dissimilarities did not produce sector-specific clades (Supplementary Figure S4). Animal and environmental pools occurred together on several branches, while the downstream pool, DS, was positioned near the two clinical pools. Pig-faecal time points, retail meat, litter, wastewater, and soil pools were distributed among multiple subclusters, indicating temporal and matrix-associated heterogeneity rather than a single livestock-associated branch. Partitioning the dendrogram into three clusters assigned both human pools to Cluster 1, animal pools to all three clusters, and environmental pools to Clusters 1 and 3 (Figure 3).

**FIGURE 3:**
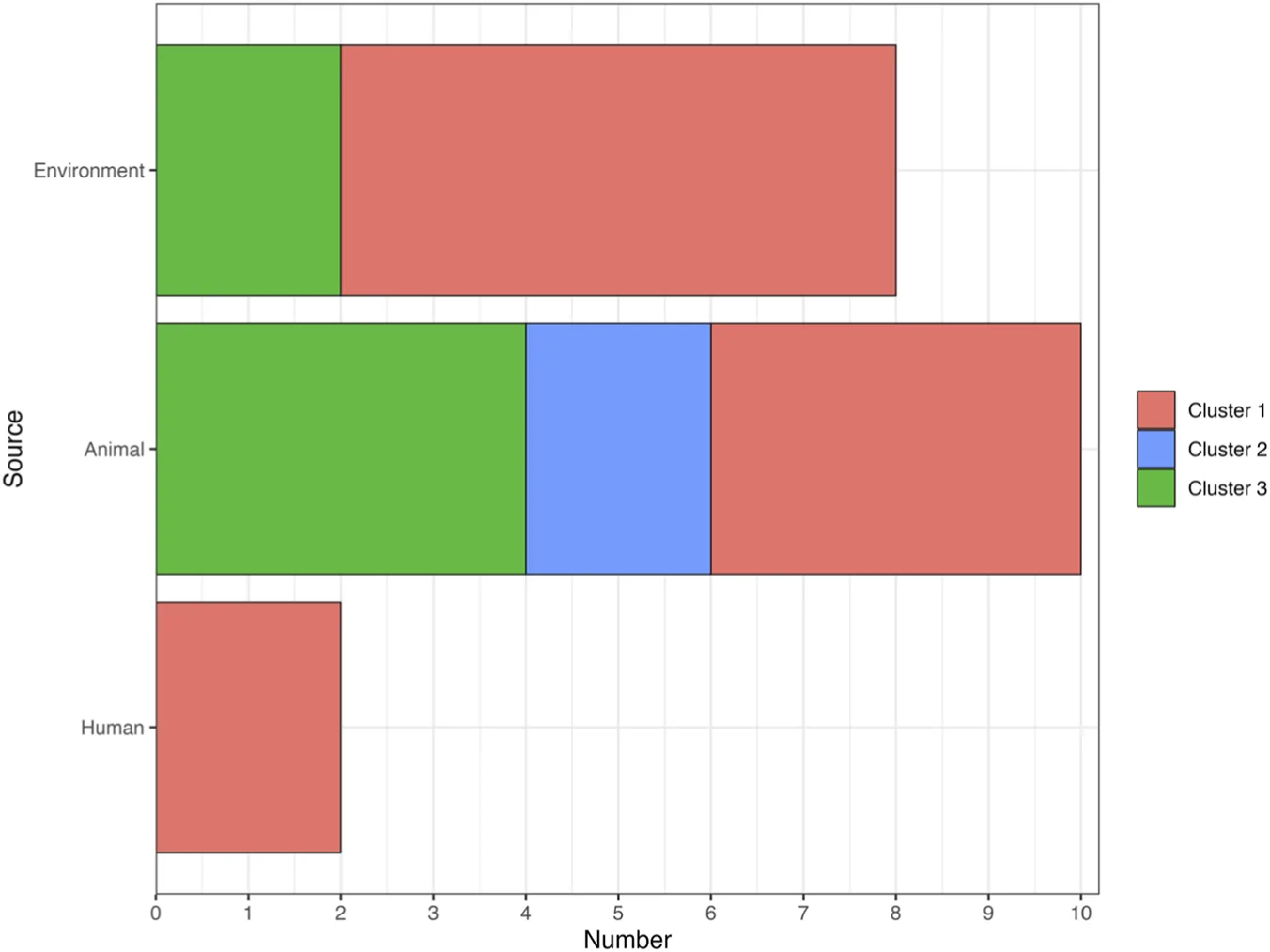
Sector distribution across resistome-profile clusters based on Bray-Curtis dissimilarity. Samples were clustered using Bray–Curtis dissimilarity computed from ARG abundance profiles. Horizontal bars indicate the number of pooled source groups from each sector (Human, Animal, Environment) assigned to Cluster 1–3 (color-coded).

Alpha diversity was comparable among the human, animal, and environmental sectors (Figure 4). Kruskal–Wallis tests detected no significant differences in observed ARG richness (χ^2^ = 0.131, *p* = 0.940), Shannon diversity (χ^2^ = 0.389, *p* = 0.820), or Simpson diversity (χ^2^ = 1.446, *p* = 0.490; all df = 2). All Dunn pairwise comparisons remained non-significant following Benjamini–Hochberg adjustment.

**FIGURE 4:**
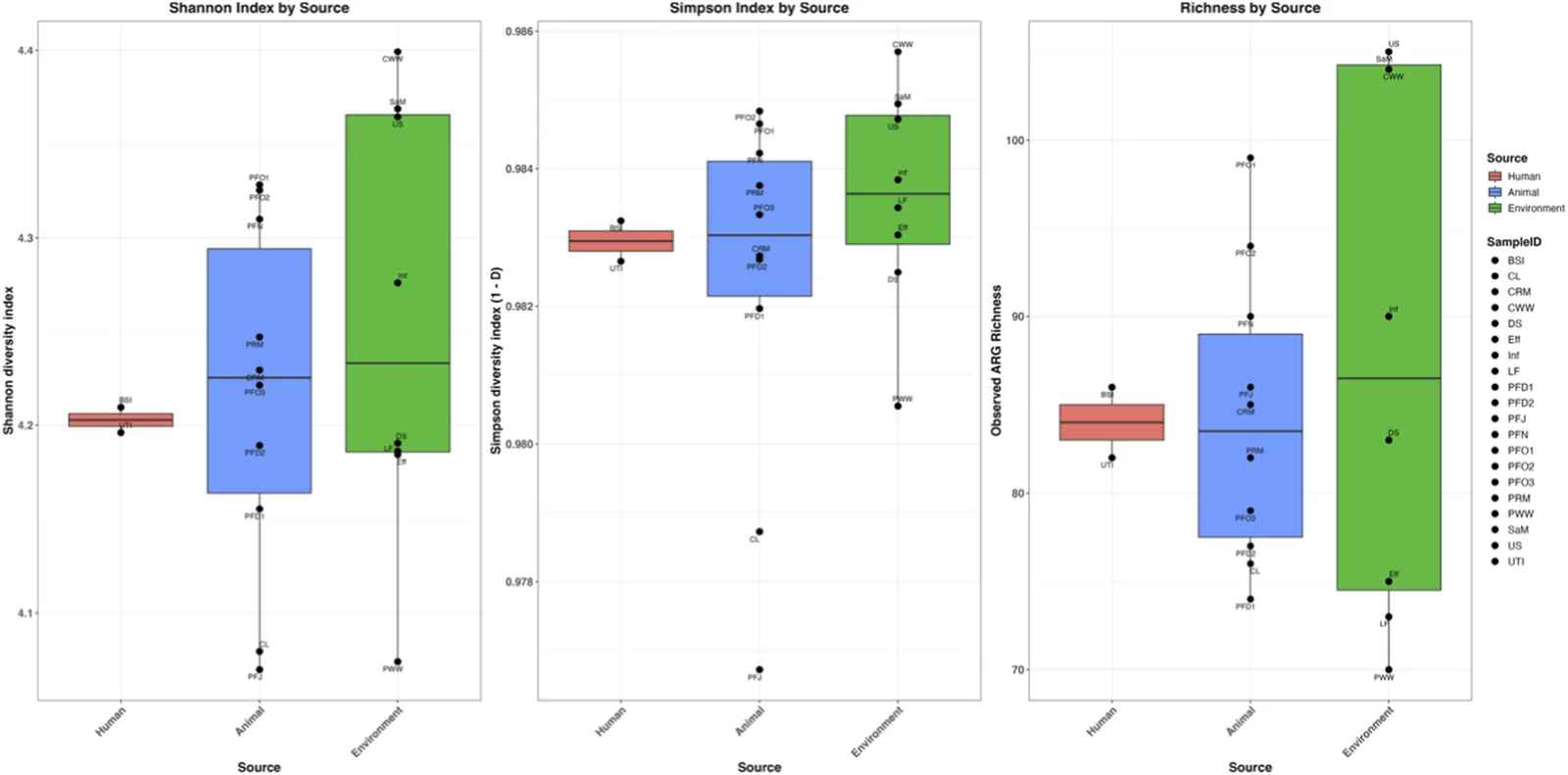
Alpha diversity metrics of *Escherichia coli* ARGs by sector computed from normalized ARG counts in an *Escherichia coli* culture metagenomic framework. Boxplots display Shannon diversity, Simpson diversity, and ARG richness; points represent individual samples, labeled by compartment.

PCoA and NMDS based on Bray–Curtis dissimilarities showed considerable overlap among sectors (Figure 5). The first two PCoA axes explained 35.1% of the variation in Bray–Curtis dissimilarities (PC1 = 20.0%; PC2 = 15.1%). Environmental pools were concentrated largely within the broader region occupied by animal pools, while animal pools exhibited the greatest dispersion, partly because of peripheral samples such as PFJ and CL. The two human pools, BSI and UTI, were positioned close to one another and near several environmental pools in both ordinations. Despite this overlap, PERMANOVA identified a statistically significant but modest association between source sector and resistome composition (pseudo-F = 1.585, *R* ^2^ = 0.157, *p* = 0.022; 999 permutations). Source sector explained 15.7% of the total variation, whereas 84.3% remained unexplained by sector. PERMDISP provided no statistically significant evidence of unequal multivariate dispersion among sectors (F_2_,_17_ = 2.498, *p* = 0.086; 999 permutations). Nevertheless, the unequal group sizes and inclusion of only two human pools warrant cautious interpretation. The clustering and ordination results indicate modest sector-associated structuring accompanied by substantial within-sector heterogeneity and cross-sector overlap. Proximity among pools reflects similarity in ARG relative-abundance profiles but does not, by itself, establish ARG exchange, transmission, epidemiological connectivity, or shared transmission pathways.

**FIGURE 5:**
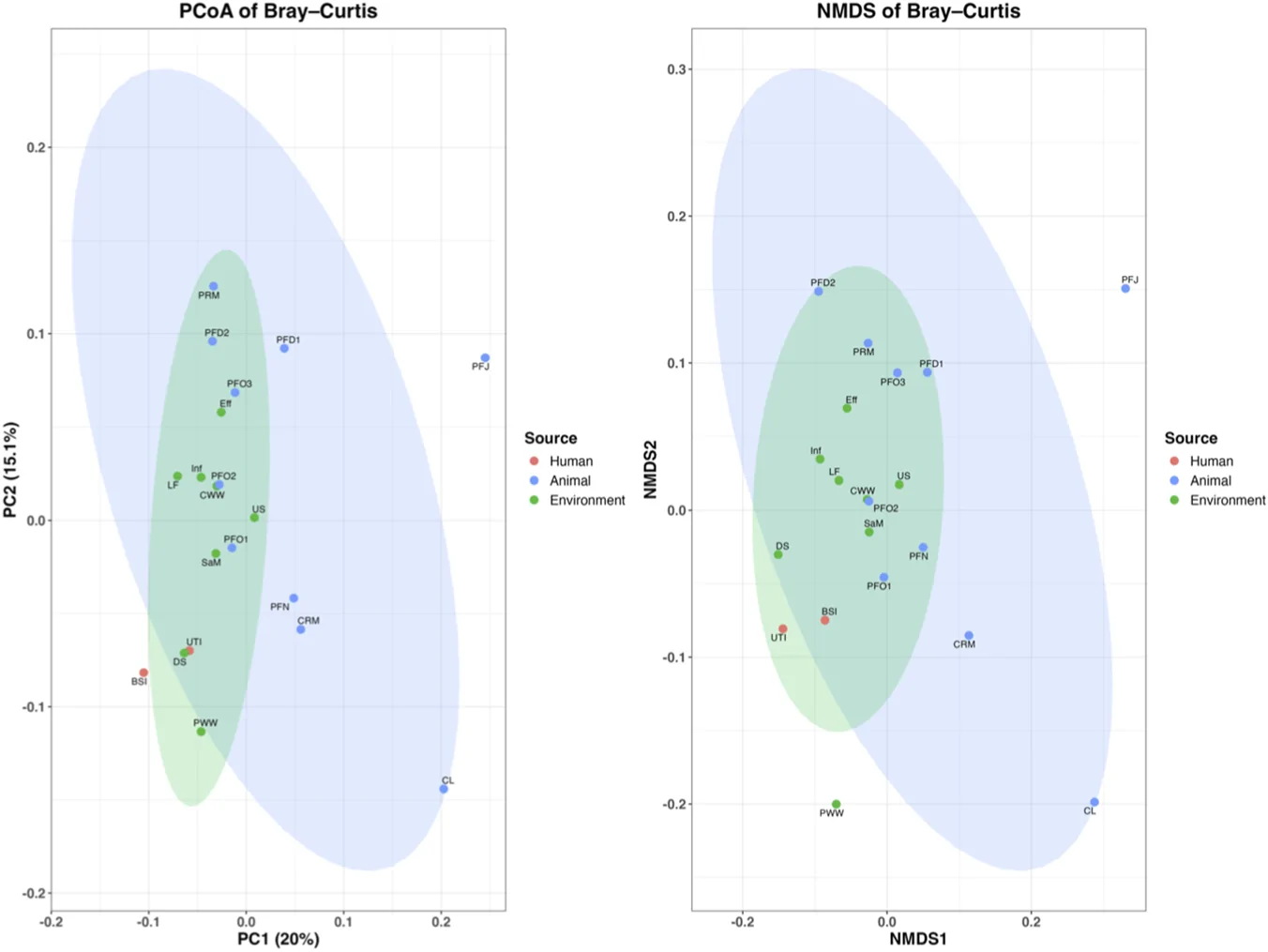
Bray-Curtis ordination of pooled *Escherichia coli* resistome profiles. The panels show PCoA and NMDS representations of ARG-profile dissimilarity. Points are labeled by pooled source group and colored by sector (human, animal, or environmental). Ellipses summarize within-sector dispersion and were displayed only for the animal and environmental sectors, which had sufficient replication. No ellipse was calculated for the human sector because it contained only two pools. Overlap indicates limited separation of relative-abundance profiles and does not establish transmission or epidemiological connectivity among sectors.

### 3.5 Resistome-risk estimates

The unweighted arithmetic mean MetaCompare score was 23.26% for the animal pools, 19.13% for the environmental pools, and 19.04% for the human pools (Supplementary Table S3). Within the human sector, the UTI pool had a higher score than the BSI pool (22% vs. 16%). Among the animal pools, chicken litter had the lowest score (13%), whereas PFJ had the highest (37%). Among the environmental pools, PWW had the lowest score (12.62%), while CWW had the highest (29.16%). Overall, the mean MetaCompare scores were broadly comparable across the three sectors, with the animal pools showing a modestly higher mean than the environmental and human pools. These MetaCompare values represent comparative prioritization scores and should not be interpreted as probabilities of clinical harm or evidence of demonstrated ARG transfer.

### 3.6 ARGs associated with resistome-profile discrimination across source groups

Exploratory Random Forest analysis identified ARG-associated features contributing to differentiation among human, animal, and environmental *E. coli* resistome profiles (Supplementary Figure S2). Repeated five-fold cross-validation yielded a best-tuned cross-validated accuracy of 63.1% (*κ* = 0.303; mtry = 2). In the tree-matched analysis, the observed cross-validated accuracy was 60.5%, compared with a mean accuracy of 47.3% under permuted source labels (permutation *p* = 0.053; Supplementary Table S4). Thus, the permutation test did not provide statistically significant evidence that classification performance exceeded chance.

Model performance and feature-ranking stability showed a mean testing accuracy of 56.2% (SD = 15.7%; range = 20.0–80.0%) and a mean out-of-bag error of 47.5% (SD = 9.1%), indicating weak discriminatory performance with considerable variability among data partitions. Feature-ranking stability was assessed by recording the frequency with which each ARG occurred among the 35 highest-ranked predictors across the 100 runs (Figure 6). *dfrA12* was the only predictor retained among the top 35 in all 100 runs. *Ecol_emrE*, *mdtN*, and *tet(D)* were retained in 99% of runs, followed by *pmrF* in 98%, *patA* and *qacH* in 95%, *sdiA* and *mdtM* in 94%, and *evgA* in 88% of runs. These recurrently selected ARGs represent an internally stable component of the fitted feature-ranking pattern.

**FIGURE 6:**
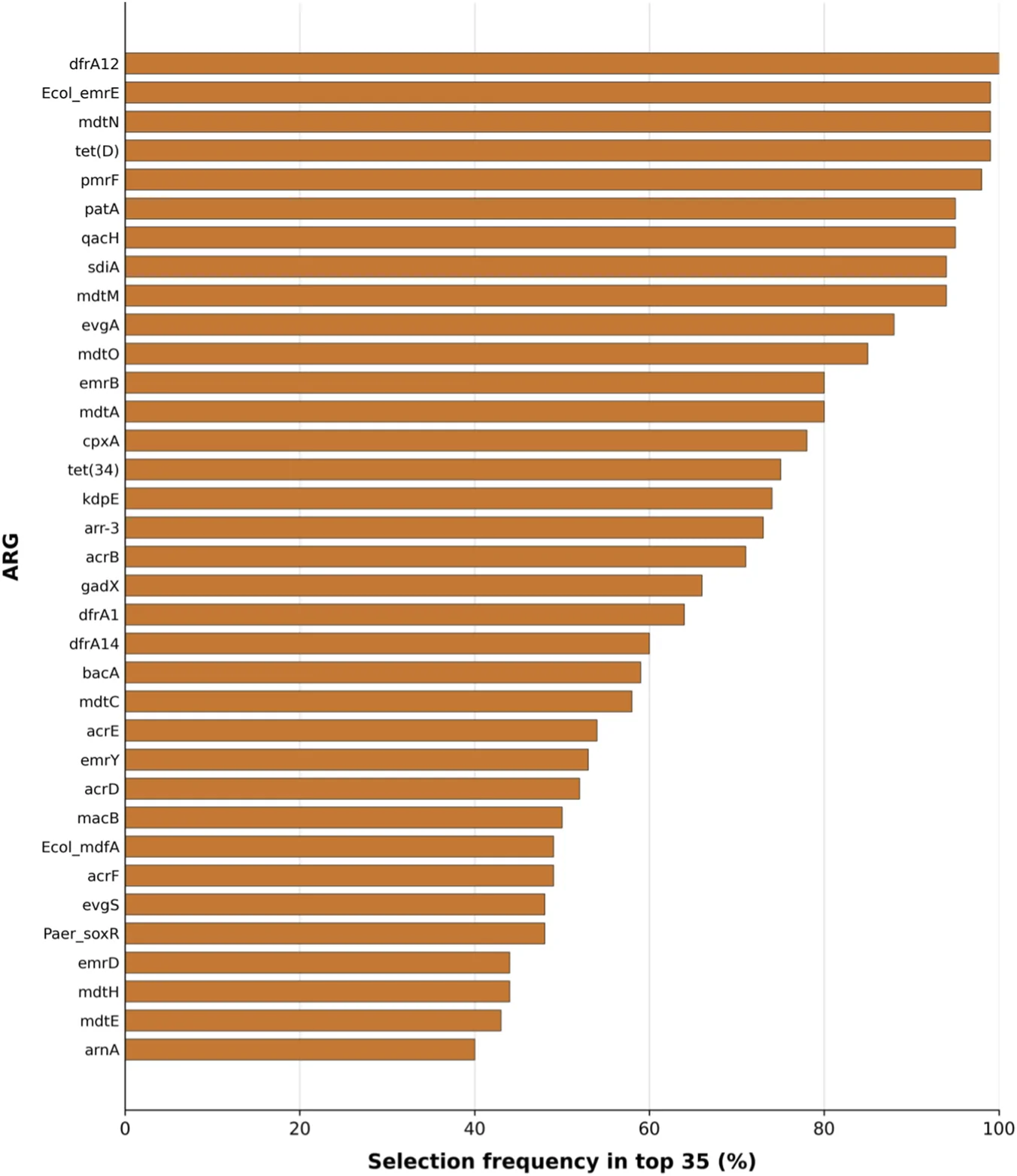
Stability of leading ARG predictors across repeated Random Forest analyses. Feature-ranking stability was evaluated across 100 repeated stratified Random Forest runs. During each run, the 20 pooled source-group profiles were partitioned into training and testing sets, a 1000-tree Random Forest was fitted, and the 35 ARGs with the highest Mean Decrease Gini values were retained. Bars represent the percentage of runs in which each ARG appeared among the top 35 predictors. Higher percentages indicate greater internal stability of the feature-ranking pattern.

### 3.7 Plasmid-associated and mobile genetic element features

Across the analyzed pools, 1,012 distinct plasmid-associated features were detected. Of these, 713 (70%) were detected in at least one analyzed pool from each sector (Supplementary Figure 3b), indicating a shared plasmid-associated feature repertoire across sectors. These matches do not represent reconstructed complete plasmids and do not demonstrate that the corresponding sequences occurred on functional or transferable plasmids. Sector-exclusive features were uncommon, comprising one human-only feature, 11 animal-only features, and one environment-only feature. A further 285 features (28%) were detected in animal and environmental pools but not in human pools, while one feature (<1%) was detected in human and environmental pools but not in animal pools. No features were detected exclusively in the human and animal pools.

Across the analyzed pools, 975 distinct MGE-associated database features were detected, including matches annotated as transposases, integrases, recombinases, and transposon- or plasmid-associated markers. Of these, 232 (24%) were detected in at least one analyzed pool from each sector (Supplementary Figure 3c). The largest pairwise-exclusive intersection comprised 267 features (27%) detected in animal and environmental pools but not in human pools. Human and environmental pools shared 15 features (2%) in the absence of animal detection, whereas human and animal pools shared three features (<1%) in the absence of environmental detection. Features uniquely detected within the analyzed pools comprised 205 environment-only (21%), 239 animal-only (25%), and 14 human-only (1%) features (Supplementary Tables S6–S7).

## 4 Discussion

AMR is a complex, multisectoral problem shaped by antimicrobial use and the persistence and movement of resistant bacteria and resistance determinants across humans, animals, and the environment. Addressing these interconnected dimensions requires a One Health approach that considers both sector-specific pressures and the interfaces through which resistant organisms and their genetic determinants may circulate (Collignon and McEwen, 2019). In this study, pooled MDR *E. coli* profiles from all three sectors showed substantial resistome overlap, although permutational multivariate analysis of variance identified a significant but modest association between sector and resistome composition. Sector explained 15.7% of the observed variation, indicating that source sector influenced resistome structure but did not produce clearly separated human, animal, and environmental profiles.

This substantial overlap is consistent with findings from sympatric humans, livestock, and peri-domestic wildlife in Nairobi, where AMR gene-community composition was not strongly associated with host species (Muloi et al., 2022). The shared profiles may reflect common resistance determinants, related *E. coli* backgrounds, similar selective pressures, comparable ecological conditions, or independent acquisition from common sources. However, the present data cannot distinguish among these explanations. Shared ARGs should therefore be interpreted as evidence of common resistance potential rather than proof of transmission between sectors. Wastewater-associated pools contributed to the observed cross-sector overlap, but the culture-based pooled design cannot determine ARG removal during treatment, persistence in receiving environments, or downstream dissemination. Previous whole-metagenome studies have reported shared ARGs between wastewater influent and effluent and variable treatment effects among resistance mechanisms (Majeed et al., 2021; Garner et al., 2024). These findings provide environmental context but should not be directly equated with the present analysis of cultured *E. coli* isolates. CARD-based detection included acquired ARGs as well as intrinsic, chromosomal, regulatory, and efflux-associated determinants. Therefore, the total resistome may reflect both acquired resistance and the underlying *E. coli* genomic background. Although separating acquired from chromosomal determinants improved interpretation, sequence detection alone cannot confirm gene expression, phenotypic resistance, genomic context, ARG–MGE linkage, or horizontal transfer. These properties require isolate-resolved genomic, phenotypic, transcriptomic, and functional analyses (Sivalingam et al., 2020; Drane et al., 2024). The findings indicate a shared but heterogeneous *E. coli* resistome across the sampled One Health sectors. The modest sector effect suggests that bacterial lineage, local exposure history, environmental conditions, and sampling factors may explain more variation than sector classification alone. Thus, the results identify cross-sector resistance patterns but do not establish transmission pathways or epidemiological connectivity.

The acquired-ARG analysis distinguished potentially horizontally acquired determinants from the broader chromosomal and intrinsic *E. coli* resistome. Acquired ARGs occurred in human, animal, and environmental pools and represented several mechanisms, including antibiotic inactivation, target protection, target replacement, acquired efflux, and target alteration. This diversity suggests that the acquired resistome was shaped by multiple resistance processes rather than one dominant mechanism. However, detection across sectors does not demonstrate transfer and may reflect common determinants, related bacterial backgrounds, similar selective pressures, or independent acquisition. The occurrence of *vgaC* and *msrB* indicates the presence of ABC-F ribosomal-protection-associated resistance determinants. These proteins can protect the ribosome from ribosome-targeting antimicrobials (Sharkey et al., 2016; Wilson, 2016; Crowe-McAuliffe et al., 2021). However, the present sequence data cannot confirm their expression, phenotypic contribution, genomic context, or transferability. The representation of *tet(D)* and *tet(34)* emphasizes the importance of tetracycline resistance within the acquired component. This finding is consistent with the frequent occurrence of tetracycline resistance determinants in livestock-associated and environmental resistomes (Checcucci et al., 2020; Lawther et al., 2022). Nevertheless, their distribution may reflect direct tetracycline selection, co-selection by other compounds, persistence of resistant lineages, or repeated introduction from common sources. The detection of *qacH* is relevant because disinfectant- and antiseptic-associated resistance may persist alongside antibiotic resistance under shared selective conditions (Boyce, 2023; Lu et al., 2024). However, this study did not measure biocide exposure or demonstrate phenotypic disinfectant tolerance. Therefore, *qacH* should be interpreted as evidence of disinfectant-associated resistance potential rather than proof of co-selection. Aminoglycoside-modifying genes, including *aadA*, APH, and AAC variants, together with *dfrA* and *sul* determinants, broadened the acquired resistance repertoire. These genes are often associated with integrons and other mobile contexts (Antunes et al., 2005; Partridge et al., 2018; Bhat et al., 2023). Their cross-sector detection may indicate shared resistance platforms, but the present analysis did not establish integron location, physical linkage, plasmid carriage, or transfer. The acquired-ARG profiles indicate diverse resistance potential across the three sectors. However, classification as acquired does not prove recent acquisition, mobility, expression, or horizontal transfer. Isolate-resolved sequencing, long-read plasmid reconstruction, phenotypic susceptibility testing, and contig-level ARG–MGE analysis are needed to determine the biological and epidemiological significance of these determinants.

Plasmid- and MGE-associated features were detected in human, animal, and environmental pools. Notably, 713 of 1,012 plasmid-associated features and 232 of 975 MG E-associated features occurred in at least one pool from each sector. The animal–environment intersection also showed the greatest pairwise overlap for ARGs, plasmid-associated features, and MGE-associated features. These findings suggest a shared resistance-feature repertoire between animal and environmental pools, but do not demonstrate genetic exchange or transmission. The animal–environment overlap is biologically plausible because livestock, litter, soil, wastewater, and drainage systems may be connected through fecal material, water, resistant bacteria, antimicrobial residues, and genetic material. Plasmids, integrons, transposons, and insertion sequences can contribute to horizontal ARG transfer among bacterial lineages (Partridge et al., 2018; Oh et al., 2018). However, the database-matched features detected here were not reconstructed as complete mobile elements. The analysis did not establish ARG–MGE co-location on the same contig, plasmid, chromosome, or bacterial strain. It also did not demonstrate gene expression, active mobility, transferability, or transmission direction. Shared features may instead reflect common bacterial backgrounds, widespread sequence markers, similar selective pressures, repeated independent acquisition, or exposure to common upstream sources. Therefore, the observed overlap should be interpreted as shared resistance potential rather than confirmed intersectoral transmission. The pronounced animal–environment overlap is consistent with evidence that livestock-associated and environmental resistomes may share resistance determinants (Lawther et al., 2022). Nevertheless, the cross-sectional pooled design cannot determine whether resistance moved from animals to the environment, from the environment to animals, or from a common unmeasured source. Isolate-resolved sequencing, long-read plasmid reconstruction, phylogenomics, and contig-level ARG–MGE analysis are required to distinguish these possibilities. Overall, the animal–environment interface represents a priority for targeted One Health surveillance, but neither sector can be identified as a confirmed source, reservoir, hub, or transmission route based on these data alone.

MetaCompare provided a comparative prioritization of resistome profiles based on associations among ARGs, MGEs, and pathogen-associated sequences (Oh et al., 2018). Mean scores were broadly similar across sectors, although animal pools had a modestly higher mean than human and environmental pools. These scores should be interpreted as comparative resistome-risk indicators rather than probabilities of clinical harm, active ARG mobility, or transmission. Variation among individual pools was more informative than sector-level averages. PFJ among animal pools and CWW among environmental pools showed comparatively elevated scores, identifying them as candidates for targeted follow-up. However, the results do not establish that these source groups were reservoirs, transmission hubs, or sources of AMR. MetaCompare scores may reflect differences in feature composition, database associations, or pooled isolate representation.

Random forest classification provided limited evidence for reliable sector discrimination. Observed cross-validated accuracy was 60.5%, compared with 47.3% after permutation of sector labels, and the difference was not statistically significant (*p* = 0.053). Mean testing accuracy across repeated partitions was 56.2%, indicating weak and variable classification performance. Thus, the model did not provide validated source attribution. Several features were repeatedly ranked among the strongest predictors, including *dfrA12*, *Ecol_emrE*, *mdtN*, and *tet(D)*, with other recurrent features including *pmrF*, *patA*, *qacH*, *sdiA*, *mdtM*, and *evgA*. Their recurrence suggests that the model used a combination of acquired ARGs and broader efflux-, regulatory-, envelope-, and stress-response-associated features. These findings must be considered in relation to the 20 independent pooled profiles and 102 informative predictors included in the model. The high predictor-to-observation ratio, unequal sector representation, limited human-sector replication, and correlations among ARGs may produce unstable or distributed importance estimates. Furthermore, Gini-based importance reflects a feature’s contribution within the fitted model and does not measure its abundance, prevalence, statistical significance, causal importance, or epidemiological relevance. Although recurrence across repeated runs demonstrates internal ranking stability, it does not constitute external validation. The identified features should therefore be treated as candidate source-associated predictors requiring evaluation in larger, balanced, independent, and isolate-resolved datasets.

Several design limitations define the scope of these findings. Isolate loss, variable resuscitation success, and differences in source recovery resulted in unequal pool sizes (3–46 colonies) and uneven sector representation, with pig-associated groups overrepresented because of the design of the original farm-to-fork study and only two human pools available. The pooled profiles, rather than the contributing isolates, were the independent analytical units. Moreover, culture and pooling produced composite *E. coli* genomic profiles that may disproportionately represent dominant strains or resistance determinants. The results therefore cannot provide unbiased whole-metagenomic abundances or isolate- and sector-level prevalence estimates. Although rank- and permutation-based methods were used and PERMDISP did not detect significant dispersion differences, the small, unbalanced design limits statistical power and generalizability. The analytical outputs are also constrained by their sequence-based and comparative nature. Database annotations cannot confirm taxonomic origin, gene expression, phenotypic resistance, complete mobile elements, or physical ARG–MGE linkage. MetaCompare and Random Forest provided exploratory prioritization rather than estimates of clinical risk or validated source attribution. Accordingly, the shared features and modest sector-associated structuring identified here should be interpreted as pooled source-group patterns that generate hypotheses for further investigation, not as evidence of prevalence, horizontal transfer, epidemiological connectivity, transmission pathways, or transmission direction.

## 5 Conclusion

This study demonstrates that MDR *E. coli* from human, animal, and environmental sources in uMgungundlovu District harbour a broadly shared but heterogeneous resistome, superimposed on modest sector-associated compositional differences. The predominance of chromosomal and intrinsic determinants suggests that background *E. coli* resistance architecture strongly shaped the observed profiles, while the cross-sector detection of acquired ARGs and plasmid- and MGE-associated features highlights a potentially important reservoir of mobilizable resistance. These findings indicate that resistance patterns are not confined to individual One Health sectors but likely reflect a combination of widespread determinants, related bacterial backgrounds, shared selective pressures, and source-specific influences. These results support integrated surveillance across human, animal, and environmental interfaces. However, determining whether the observed similarities arise from clonal dissemination or horizontal gene transfer will require balanced longitudinal sampling, individual-isolate sequencing, phenotypic testing, phylogenomics, contig-level ARG–MGE analysis, and long-read plasmid reconstruction.

## Data Availability

The data presented in this study are deposited in the NCBI Sequence Read Archive (SRA) under BioProject accession number PRJNA1431768 (https://www.ncbi.nlm.nih.gov/bioproject/PRJNA1431768). All other data supporting the findings of this study are included in the article and its Supplementary Material.
